# Maresin 1 Mitigates Sepsis-Associated Acute Kidney Injury in Mice *via* Inhibition of the NF-κB/STAT3/MAPK Pathways

**DOI:** 10.3389/fphar.2019.01323

**Published:** 2019-11-07

**Authors:** ShuJun Sun, JiaMei Wang, JingXu Wang, FuQuan Wang, ShangLong Yao, HaiFa Xia

**Affiliations:** ^1^Department of Anesthesiology, Union Hospital, Tongji Medical College, Huazhong University of Science and Technology, Wuhan, China; ^2^Institute of Anesthesia and Critical Care Medicine, Union Hospital, Tongji Medical College, Huazhong University of Science and Technology, Wuhan, China; ^3^College of Life and Health Sciences, Northeastern University, Shenyang, China

**Keywords:** Maresin 1, Sepsis, acute kidney injury, inflammatory, cecal ligation and puncture

## Abstract

Acute kidney injury (AKI) is one of the most common and serious complications of sepsis in which the inflammatory cascade plays a crucial role. There is now increasing evidence that lipid mediators derived from the omega-3 fatty acid docosahexaenoic acid (DHA) have potent anti-inflammatory effects that promote the timely regression of acute inflammation. In this study, we investigated the protective effects and molecular mechanism of a novel DHA-derived lipid mediator Maresin 1 (MaR1) on AKI in septic mice. The cecal ligation and puncture (CLP) was used to establish a sepsis mice model. As a result, we found that MaR1 significantly increased the 7-day survival rate of septic mice and the anti-inflammatory factor IL-10 while reducing bacterial load and pro-inflammatory cytokines (TNF-α, IL-6, and IL-1β). In addition, MaR1 dose dependently reduced renal injury scores and serum creatinine and urea nitrogen levels in septic mice while inhibiting renal neutrophil infiltration and myeloperoxidase (MPO) activity. In terms of signaling pathway, we found that MaR1 inhibits the expression of phosphorylated p65, Stat3, JNK, ERK, and p38 and significantly reduces nuclear translocation of p65. In conclusion, our results indicate that MaR1 is able to reduce neutrophil infiltration and inhibit nuclear factor-kappa B/signal transducer and activator of transcriptor 3/mitogen-activated protein kinase (NF-κB/STAT3/MAPK) activity and regulate inflammatory cytokine level to inhibit inflammatory response and thereby weaken sepsis-associated AKI in mice.

## Introduction

Sepsis is a life-threatening clinical syndrome whose characteristics and high mortality rates have been known for centuries, but a unified and well-defined definition has only appeared for decades (Poston and Koyner, 2019). The recent definition of sepsis revised in 2016, which defined sepsis as “life-threatening organ dysfunction caused by a dysregulated host response to infection,” eliminating systemic inflammatory response syndrome (SIRS) and severe sepsis, and marking that definition of sepsis from the continuum of SIRS, sepsis, severe sepsis, and septic shock were further refined (Bone et al., 1992; Levy et al., 2003; Singer et al., 2016). Regardless of how the definition of sepsis is revised, it is an indisputable fact that the incidence of sepsis is rising in the intensive care unit (ICU) and the mortality associated with severe sepsis remain high (Doi et al., 2009; Chen et al., 2014). Sepsis can often progress to multiple organ failure, and acute kidney injury (AKI) is one of the most common and serious complications in the development of sepsis, which is characterized by inadequate blood filtration, imbalance of water and ions, and impaired urine production (Mehta et al., 2007; Chen et al., 2014). Studies have shown that sepsis-associated AKI (SA-AKI) accounts for 26% to 50% of all AKI in developed countries, while primary nephropathy-related AKI only 7% to 10% (Bagshaw et al., 2008; Alobaidi et al., 2015). More importantly, the hospital mortality in patients with sepsis developing AKI will increase six to eight times, and even if the patients survived, the risk of progression to chronic renal failure is significantly increased (Gómez and Kellum, 2016). However, unfortunately, the pathophysiological mechanism of AKI caused by sepsis is not fully understood, but it can be confirmed that the harmful inflammatory cascade of sepsis plays an important role in the occurrence and development of AKI (Ricci and Ronco, 2009; Poston and Koyner, 2019).

In recent years, endogenous anti-inflammatory and pro-resolving bioactive mediators derived from omega-3 polyunsaturated fatty acids (PUFAs) have received widespread attention. These fatty acids and their oxidized derivatives are widely distributed in organisms, such as protectins and resolvins, which have strong anti-inflammatory and pro-resolving effects (Serhan et al., 2002; Serhan, 2006). Maresin 1 (MaR1) is a newly discovered endogenous lipid mediator, the first chemically isoform of maresins, synthesized by macrophages using docosahexaenoic acid (DHA) (Buckley et al., 2014). Recent studies have found that MaR1 attenuates kidney and cerebral ischemia/reperfusion injury by inhibiting inflammation, oxidative stress and apoptotic pathway in the mice model of ischemia–reperfusion, and promotes inflammation regression and thereby reduces acute lung injury by inhibiting neutrophil infiltration and enhancing macrophage phagocytosis in the mice model of sepsis acute lung injury (Gong et al., 2015; Qiu et al., 2019; Xian et al., 2019). In addition, MaR1 exhibits significant anti-inflammatory and pro-resolving effects in multiple mice models such as postoperative neurocognitive disorders, rheumatoid arthritis, and atherosclerosis (Viola et al., 2016; Jin et al., 2018; Yang et al., 2019). However, whether or not MaR1 has the same protective effect in the SA-AKI mice model and its related mechanism has not been reported yet. In view of the previous literature on MaR1, we predicted that MaR1 intervention could inhibit the inflammatory response of SA-AKI and reduce renal tissue damage.

## Materials and Methods

### Experimental Animals

Male C57BL/6 mice (6–8 weeks, weight 20–25 g) were purchased from Experimental Animal Center of Wuhan University, Wuhan, China (Laboratory Animal Certificate: SCXK 2014-0004). The mice were housed under standard temperature and humidity, light/dark cycles for 12 h, and adequate food and water. All animal-related experimental procedures were conducted under the National Institutes of Health (NIH) guidelines and were approved by the Animal Committee of Tongji Medical College, Huazhong University of Science and Technology (Wuhan, China).

### Animal Model Preparation and Drug Administration

The mice were randomly divided into four groups: sham-operated group (Sham), cecal ligation and puncture group (CLP), MaR1 low-dose group (LD-MaR1), and MaR1 high-dose group (HD-MaR1). The sepsis model was prepared by the CLP method (Rittirsch et al., 2009). First, we used 2% sodium pentobarbital to anesthetize mice by intraperitoneal injection at 80 mg/kg. Then, we placed the mice in the supine position on the laboratory bench, and we performed skin preparation and disinfection of the operating area. Next, we created a 1.5-cm incision in the midline of the abdomen of the mice and dissociated and fully exposed the cecum. And then, we used a sterile sewing silk no. 4 to ligature the cecum at 1 cm away from the cecum tail, and we used a 20-gauge needle to perforate the blind end and squeezed a little feces. Finally, we restored the cecum to the abdominal cavity and sutured the abdominal incision layer by layer. After the surgery, 1 ml of normal saline (NS) was injected subcutaneously into the back of the mice for liquid resuscitation, and the mice were placed in a thermostatic blanket for rewarming. The sham group was operated as before, but not cecal ligation and perforation.

MaR1 was purchased from Cayman Chemical (Ann Arbor, MI, USA). At 1 h after CLP, the LD-MaR1 group was injected with MaR1 0.5 ng which was diluted to 200 µl of sterile NS *via* the tail vein, the HD-MaR1 group was injected with MaR1 1 ng diluted to the same volume with NS *via* the tail vein, and the sham group and the CLP group were injected with the same volume of NS.

### Survival Analysis and Animal Specimen Collection

The mice (12 in each group) underwent surgery and drug treatment as described above, and the number of mice deaths within 7 days were observed and recorded every 24 h after administration for survival analysis, and the mortality rate was calculated during the observation period.

Parallel experiments were performed for specimen collection. The mice were moderately anesthetized 24 h after CLP. Whole blood was taken from one eyeball (500∼1,000 µl of whole blood was taken from each mice), and it was placed at room temperature for 1 h and then centrifuged at 3,000 rpm for 10 min. The supernatant was placed in an EP tube and stored in a −80°C refrigerator for ELISA and renal function analysis. Then the operator opens the abdominal cavity along the midline of the abdomen, separates and exposes the aorta and kidney, cuts the abdominal aorta and bleed thoroughly, removes the left kidney and fixes it in 4% formaldehyde for pathological examination, and takes the right kidney into the EP tube and stored in a −80°C refrigerator for subsequent testing.

### Colony-Forming Unit of Peritoneal Lavage Fluid and Blood

After 24 h of CLP, the mice were moderately anesthetized and fixed in a clean bench. The operator gently cuts the skin on the abdomen surface to fully expose the peritoneum and absorbed 3 ml of sterile phosphate-buffered saline (PBS) with a 5-ml syringe and slowly injected into the abdominal cavity through the peritoneum. Then the peritoneal lavage fluid (PLF) was extracted after gentle peritoneum with sterile cotton swabs for bacterial culture. The same procedure was repeated three times. In addition, the mice heart was fully exposed, and about 200 µl of blood was extracted and stored in a sterile EP tube for bacterial culture. The PLF and blood were separately pipetted 50 µl and continuously diluted six times with PBS, and then 50 µl of diluted PLF and blood was uniformly coated on the trypsin soy blood agar plates. The plates were incubated at 37°C for 24 h under aerobic conditions and then colony-forming unit (CFU) was counted.

### Renal Histology Assessment

The left kidney was fixed with 4% paraformaldehyde for 24 h and then embedded in paraffin. Sections 4 µm thick were prepared from the wax blocks containing renal tissue and stained with hematoxylin and eosin (H&E) and periodic acid-Schiff (PAS) reagents. The pathological changes of mice kidney tissue were observed under ordinary light microscope. Renal injury was blindly scored by an investigator according to the renal tissue damage scoring criteria described in previous report (Yu et al., 2015).

### Immunohistochemistry Detection of Neutrophile

Paraffin sections were dewaxed by xylene and gradient alcohol and placed in an EDTA buffer for microwave repair. After natural cooling, the sections were washed with TBS for three times, 5 min each time. The sections were incubated in 3% hydrogen peroxide solution at room temperature for 25 min and then sealed with 10% goat serum for 20 min. The 50 µl of ly-6G antibody (Cell Signaling Technology, Danvers, MA, USA) dilution (1:100) was added to each section and overnight at 4°C. After rinsing with TBS, 50 µl of secondary antibody (ChemMate EnVision™+/HRP) was added to each section and incubated for 50 min at 4°C. Finally, 50 µl of DAB solution was added to the sections for color development and hematoxylin counterstaining.

### Immunofluorescence Assay

Paraffin sections are dewaxed, then permeabilized with PBS containing 1% Triton X-100 for 10 min, blocked with 10% goat serum for 1 h, and added to the diluted primary antibody (rabbit-anti P65 1:400, Cell Signaling Technology, Danvers, MA, USA) at 4°C overnight. Rinse three times with PBS for 5 min each time. After diluting the fluorescently labeled secondary antibody with antibody dilution buffer, incubate the specimen for 1–2 h at room temperature in the dark. The operator who is unclear the experimental grouping used a confocal microscopy (Olympus fv3000, Tokyo, Japan) for image acquisition.

### Renal Function Assessment

The serum creatinine (Cr) and blood urea nitrogen (BUN) levels were analyzed using Olympus AU400 automated chemistry analyzer (Olympus, Tokyo, Japan). The operator sets the parameters according to the instructions of the instrument, places the samples and reagents to be tested, and reads the data.

### Enzyme-Linked Immunosorbent Assay

The serum stored in the −80°C refrigerator was thawed and then strictly in accordance with the ELISA kits (RayBiotech, Inc. Norcross, GA, USA) instructions to detect the TNF-α, IL-6, IL-1β, and IL-10 levels in the serum. The same method was used to measure inflammatory cytokines in renal tissue homogenates. In addition, the myeloperoxidase (MPO) activities of kidney tissue homogenate were determined using an MPO test kit (Nanjing Jiancheng Bioengineering Institute, Nanjing, China) according to the manufacturer’s instructions.

### Western Blot Analysis

The kidney tissue, which was stored in the −80°C refrigerator, was thawed and placed in a glass grinder, and the lysate mixture (containing protease inhibitor and phosphatase inhibitor) was added to be fully ground to obtain a homogenate. The homogenate was lysed on ice for 30 min and centrifuged at 12,000 rpm for 15 min. The supernatant was absorbed and stored in EP tube, and then the protein concentration was determined with bicinchoninic acid (BCA) kit. A 10% sodium dodecyl sulfate polyacrylamide gel electrophoresis (SDS-PAGE) gel was prepared, and the sample was loaded and then separated by electrophoresis and transferred to a polyvinylidene fluoride (PVDF) membrane. The membranes were incubated with the following primary antibody at 4°C overnight: phosphorylation index (p-p65, p-Stat3, p-JNK, p-ERK, p-p38), non-phosphorylation index (p65, Stat3), and β-actin (Cell Signaling Technology, Danvers, MA, USA) at 1:1,000. After adequately washing on the next day, primary antibody binding was detected with a secondary antibody (Cell Signaling Technology) at 1:4,000. The enhanced chemiluminescence reagent (Beyotime Institute of Biotechnology, Shanghai, China) was added for color development, exposure imaging was performed using the UVP imaging system (Upland, CA, USA), and the absorbance values of the images were analyzed using Image J software.

### Statistical Analysis

Kaplan–Meier survival curves and log-rank statistics were used to analyze the survival rate of each group. The experimental results are expressed as mean ± standard deviation (mean ± SD) for normal distribution data while median and interquartile range (IQR) for non-normal distribution data, and statistical analysis and mapping were performed using GraphPad Prism 6.0 software. The one-way analysis of variance (ANOVA) was used for comparison among groups with normality and homoscedasticity, and then the differences between groups were compared using Student–Newman–Keuls. Otherwise, the Kruskal–Wallis was used to compare the non-normal distribution or heteroscedasticity data among groups. *P* < 0.05 was considered to be statistically significant.

## Results

In this study, we performed Shapiro–Wilk and Levene tests on the data. The results showed that the data were normal and homoscedastic among groups. We further performed the ANOVA and Student–Newman–Keuls tests on the data. The specific research results are as follows.

### MaR1 Reduced Mortality and Bacterial Load in Sepsis Mice

**Figure 1:**
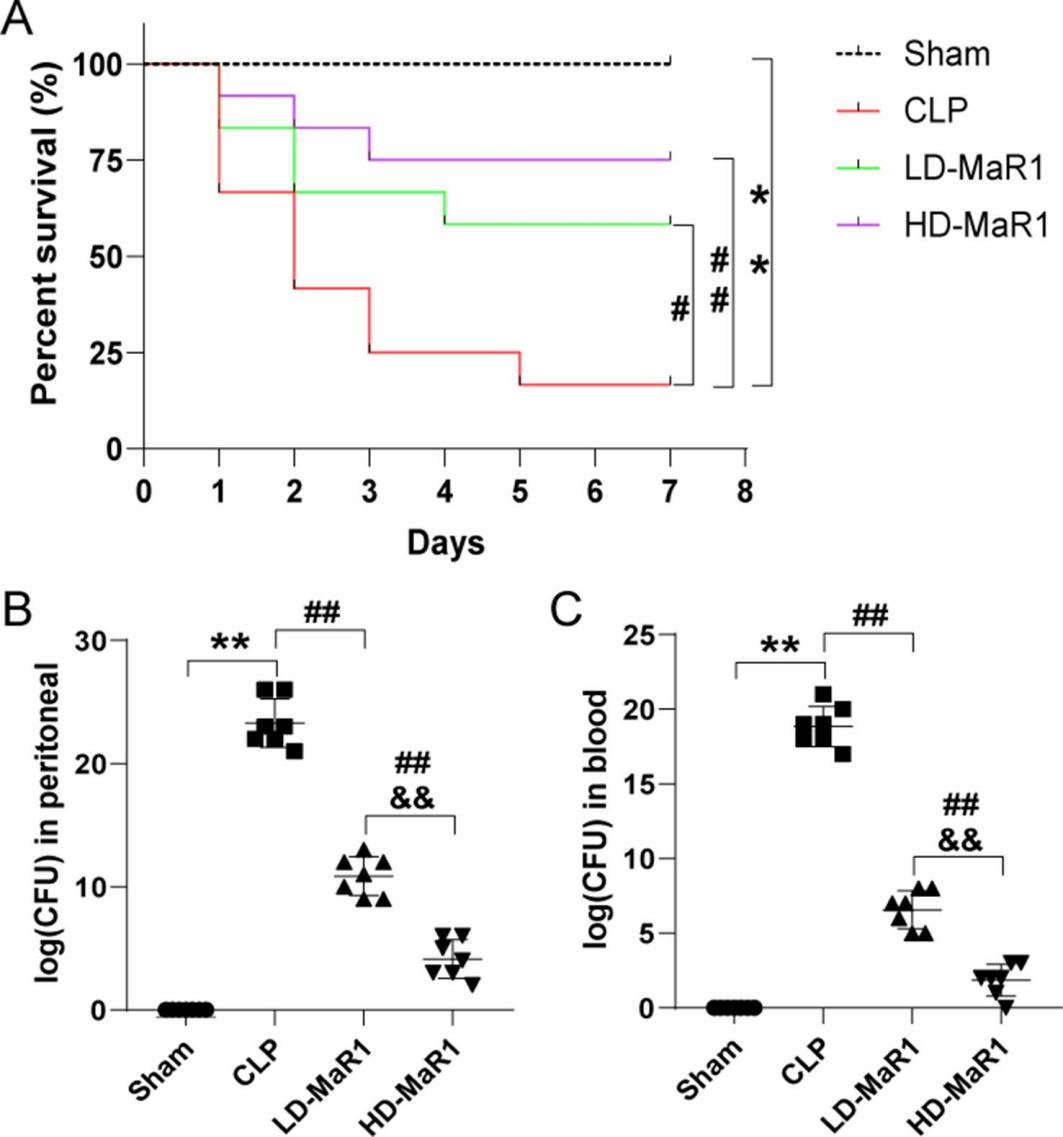
MaR1 reduced mortality and bacterial load of mice after CLP. **(A)** Kaplan–Meier survival curves showed that MaR1 significantly improved the survival rate of sepsis mice. The bacterial load in peritoneal lavage fluid **(B)** and blood **(C)** of septic mice were significantly reduced by MaR1 treatment. Data are shown as mean ± SD, *n* = 12. **P 0.01 compared to sham group; ^#^ *P* 0.05, ^##^ *P* 0.01 compared to CLP group; ^&&^ *P* 0.01 compared to LD-MaR1 group. MaR1, Maresin 1; LD-MaR1, MaR1 low-dose group.

To investigate the protective effects of MaR1 on sepsis mice, we observed the 7-day survival of the four groups of mice. As shown in **Figure 1A**, the 7-day survival rates of the sham group, the CLP group, the LD-MaR1 group, and the HD-MaR1 group were 100%, 16.67%, 58.33%, and 75%, respectively. The mortality rate was significantly increased in the CLP group compared with the sham group (*P* < 0.01). At the same time, the mortality rate of sepsis mice was significantly reduced after treatment with MaR1, either LD-MaR1 (*P* < 0.05) or HD-MaR1 (*P* < 0.01). Although the 7-day survival rate of the HD-MaR1 group was higher than that of the LD-MaR1 group, there was no statistical difference (*P* > 0.05).

We measured the amount of bacteria in the PLF and blood 24 h after CLP. As shown in **Figures 1B, C**, compared with the sham group, the CFU counts in the PLF and blood of the CLP group were significantly increased (*P* < 0.01). After treatment with MaR1, the CFU counts in the sample were significantly reduced in a dose-dependent manner (*P* < 0.01).

### MaR1 Alleviated Renal Injury of Mice After CLP

**Figure 2:**
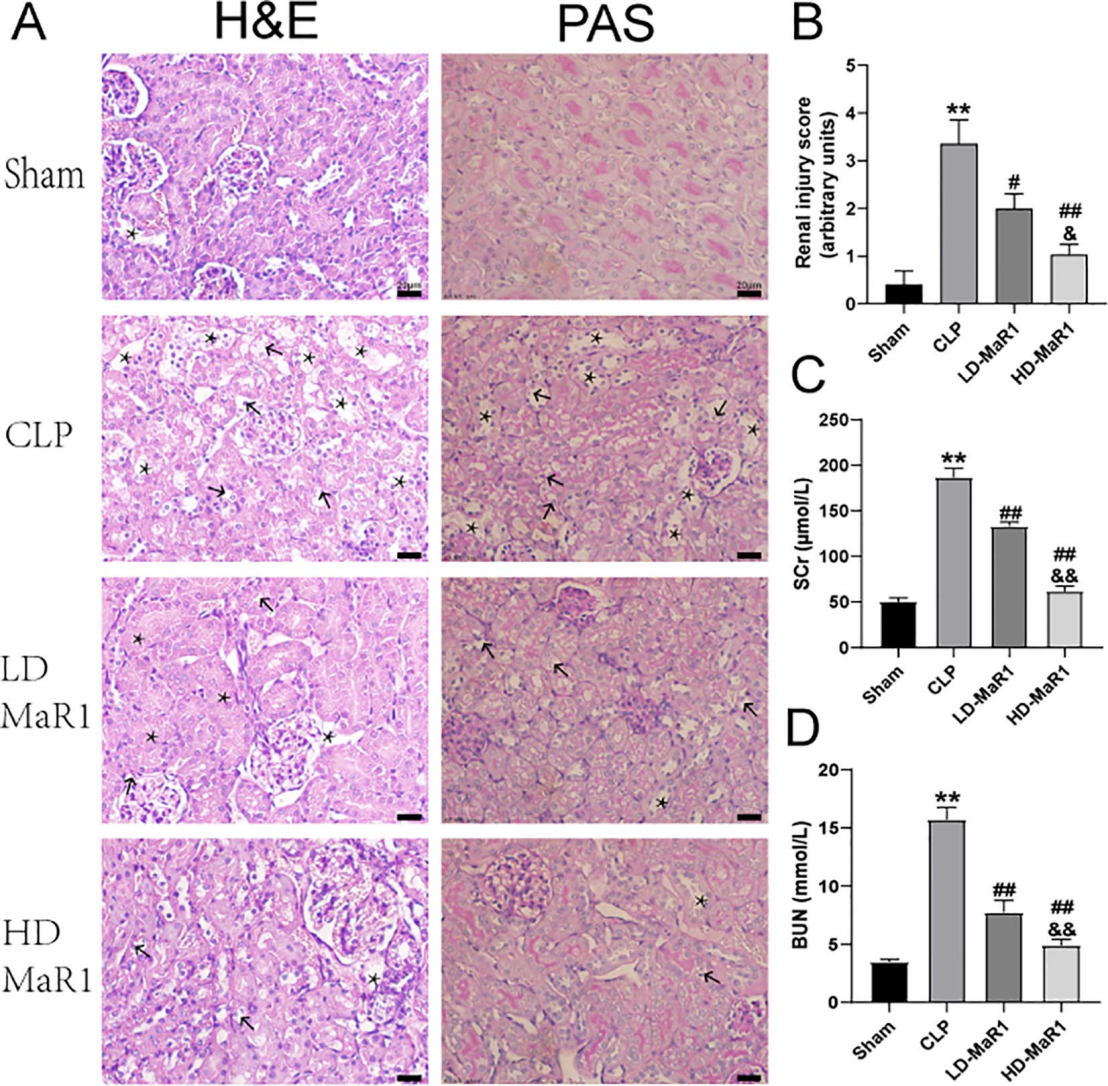
MaR1 alleviated renal injury of mice after CLP. **(A)** Representative photomicrograph of renal histology (H&E and PAS staining, magnification 400×). CLP-induced renal tissue damage with significant swelling of the renal proximal convoluted tubules, dilatation of the renal tubules and renal capsules or formation of cast (*), and renal tissue cell vacuolation (arrows). **(B)** Pathological renal tissue damage score. Serum creatinine **(C)** and blood urea nitrogen **(D)** levels at 24 h after CLP in each group of mice. Data are shown as mean ± SD, *n* = 9. **P 0.01 vs sham group; ^#^ *P* 0.05, ^##^ *P* 0.01 vs. CLP group; ^&^ *P* 0.05, ^&&^ *P* 0.01 vs. LD-MaR1 group. MaR1, Maresin 1; LD-MaR1, MaR1 low-dose group; CLP, cecal ligation and puncture; PAS, periodic acid-Schiff.

To observe the protective effects of MaR1 on SA-AKI, we performed H&E and PAS staining on kidney tissue samples (**Figure 2A**). There were no obvious abnormal pathological changes in the kidney tissue of the sham group. In the CLP group, the renal tissues were significantly edematous, the glomerular structure was not clear, the renal cystic lumen was significantly narrowed, the renal tubules were dilated and deformed, and the tubule epithelial cells shed and brush border disappeared, accompanied by a large number of inflammatory cell infiltration. The MaR1 group showed a marked reduction in renal histopathologic changes. Compared with sham group, renal injury score of the CLP group was significantly increased (*P* < 0.01), and then, compared with the CLP group, renal injury score of the MaR1 group was decreased in a dose-dependent manner (*P* < 0.05), as shown in **Figure 2B**.

We also tested the levels of blood Cr and BUN (**Figures 2C, D**). The serum Cr and BUN in CLP group were significantly higher than normal physiological range, and the difference was statistically significant compared with Sham group (*P* < 0.01). After MaR1 treatment, the levels of serum Cr and BUN of mice were decreased in a dose-dependent manner (*P* < 0.01).

### MaR1 Reduces Inflammation in Sepsis Mice

**Figure 3:**
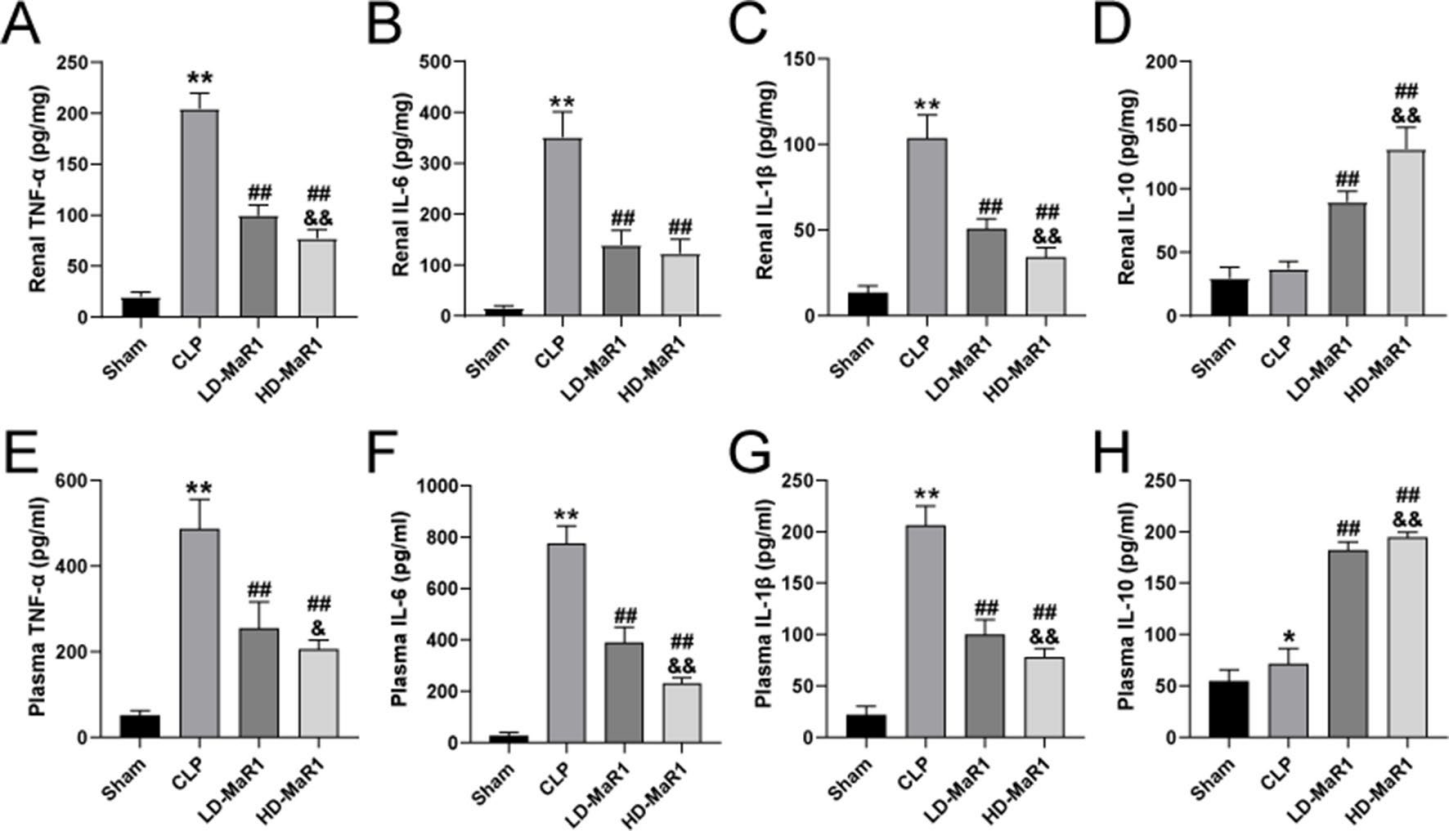
MaR1 attenuated renal and systemic inflammatory responses in sepsis mice. The TNF-α **(A, E)**, IL-6 **(B, F)**, IL-1β **(C, G)** and IL-10 **(D, H)** at 24 h after CLP in each group of mice. Data are shown as mean ± SD, *n* = 9. *P 0.05, **P 0.01 vs sham group; ^##^ *P* 0.01 vs. CLP group; ^&^ *P* 0.05, ^&&^ *P* 0.01 vs. LD-MaR1 group. MaR1, Maresin 1; CLP, cecal ligation and puncture; LD-MaR1, MaR1 low-dose group.

Inflammatory cytokines play a crucial role in inflammatory cascade and neutrophil activation and chemotaxis. The detection results of inflammatory cytokines in renal tissue homogenates are shown in **Figures 3A–D**. The level of inflammatory cytokines (TNF-α, IL-6, IL-1β, and IL-10) were low in the sham group, while the CLP group is significantly increased (TNF-α, IL-6, and IL-1β) and was statistically different from the sham group (*P* < 0.05). After treatment with MaR1, the level of pro-inflammatory cytokines (TNF-α, IL-6, and IL-1β) were significantly decreased, while the anti-inflammatory factor IL-10 was higher than that of CLP mice (*P* < 0.01). In addition, we also tested inflammatory cytokines in peripheral blood of mice to assess systemic inflammation, and the trend of results was basically consistent with the above results, as shown in **Figures 3E–H**.

### MaR1 Inhibited Renal Neutrophil Infiltration in Septic Mice

**Figure 4:**
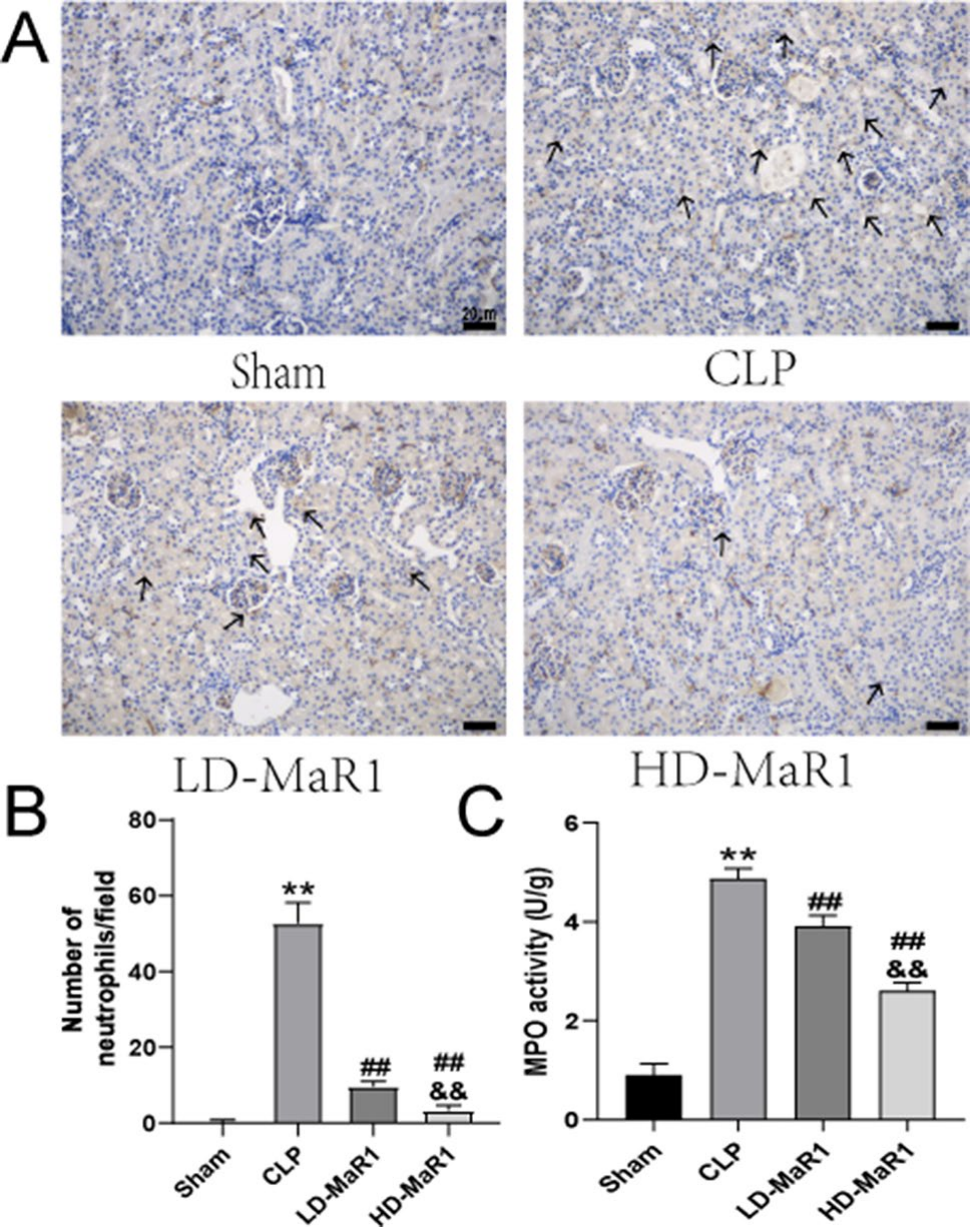
MaR1 inhibits renal tissue neutrophil infiltration and MPO activity in sepsis mice. **(A)** Representative micrographs of kidney tissue stained with neutrophil-specific Ly-6G antibody 24 h after CLP in each group of mice; black arrows indicate neutrophils, magnification 400×. **(B)** Neutrophil count/field. **(C)** MPO levels in renal tissue. Data are shown as mean ± SD, *n* = 9. **P 0.01 vs sham group; ^##^ *P* 0.01 vs. CLP group; ^&&^ *P* 0.01 vs. LD-MaR1 group. MaR1, Maresin 1; MPO, myeloperoxidase; CLP, cecal ligation and puncture; LD-MaR1, MaR1 low-dose group.

We used ly-6G immunostaining to observe renal neutrophil infiltration in septic mice. As shown in **Figures 4A, B**, the number of ly-6G cells in the CLP group was significantly increased compared to the sham group. Furthermore, MaR1 treatment resulted in a dose-dependent decrease in the number of ly-6G-positive cells, indicating that renal neutrophil infiltration was inhibited. The MPO is a function and activation marker of neutrophils, and we used MPO kit to detect the activity of MPO in renal tissue homogenate. The results showed that the MPO activity in mice kidney tissue was significantly increased after CLP. However, compared with the CLP group, MaR1 treatment significantly reduced MPO activity in a dose-dependent manner (**Figure 4C**).

### MaR1 Inhibits NF-kB/STAT3 Activation in CLP-Induced AKI

**Figure 5:**
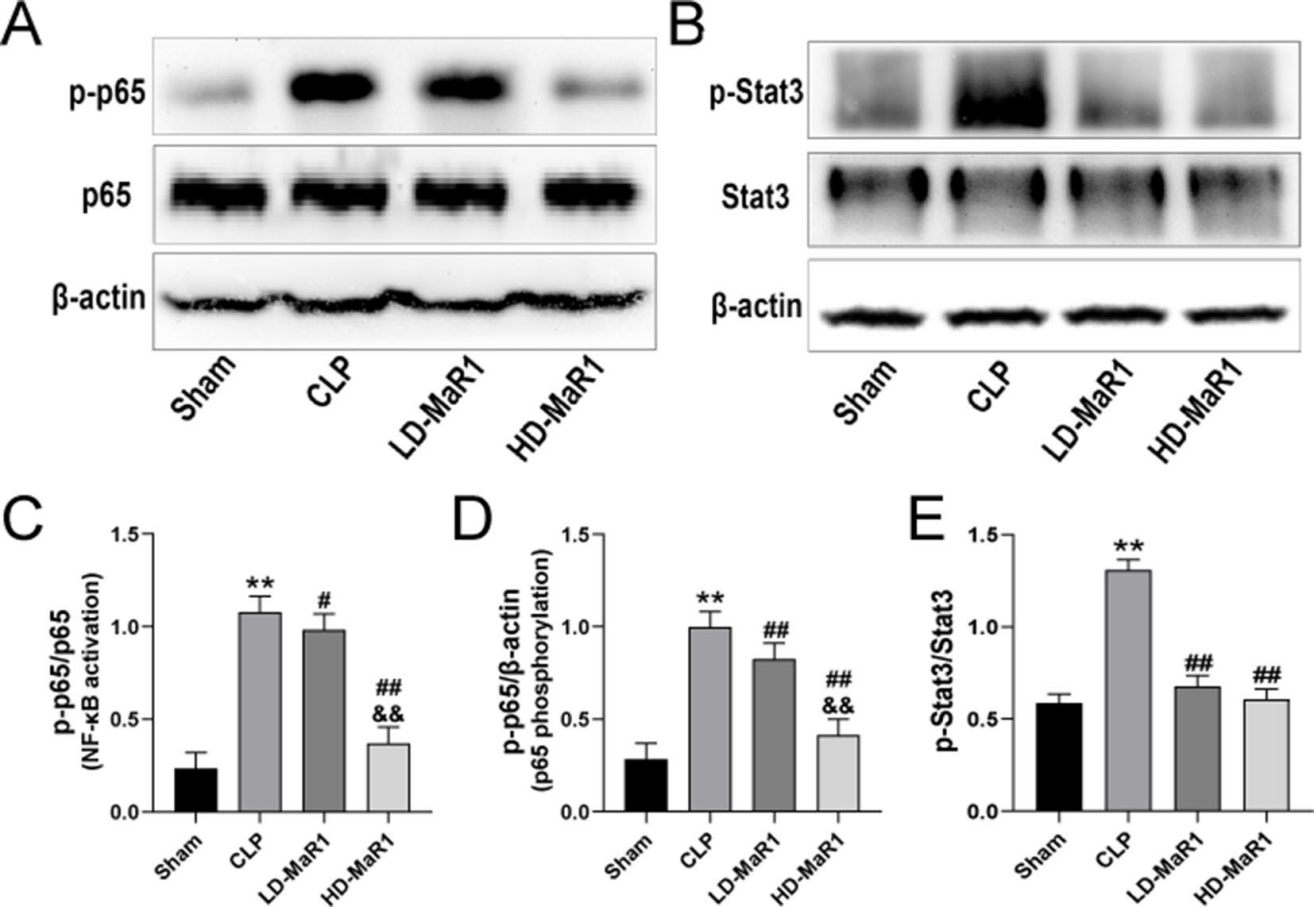
MaR1 inhibits NF-κB/STAT3 pathway activation in the kidney of sepsis mice. The expressions of p-p65, p65 **(A)** and p-Stat3, Stat3 **(B)** in renal tissues of mice in each group were analyzed by Western blot. The p-p65 protein levels normalized by p65 **(C)**, p-p65 protein levels normalized by β-actin **(D)**, p-Stat3 protein levels normalized by Stat3 **(E)**. Data are shown as mean ± SD, *n* = 9. **P 0.01 vs. sham group; ^#^ *P* 0.05, ^##^ *P* 0.01 vs. CLP group; ^&&^ *P* 0.01 vs LD-MaR1 group. MaR1, Maresin 1; NF-κB, nuclear factor-kappa B; STAT3, signal transducer and activator of transcriptor 3; CLP, cecal ligation and puncture; LD-MaR1, MaR1 low-dose group.

The nuclear factor-kappa B (NF-κB) signaling pathway mediates the expression of a variety of pro-inflammatory mediators and plays an important role in the amplification of inflammation. And the signal transducer and activator of transcriptions (STATs) proteins are widely expressed in different types of cells and tissues and play an important role in IL-6-mediated inflammatory signaling pathways. To further explore the protective mechanism of MaR1 in SA-AKI, we analyzed the activation of the nuclear translocation of NF-κB p65 and STAT3 in kidney tissue. As can be seen from **Figures 5A–E**, the phosphorylation level of p65 and STAT3 in the kidney tissue of the sham group was low, while the CLP group significantly increased and was statistically significant compared with the sham group (*P* < 0.01). After MaR1 intervention, the phosphorylation level of p65 and STAT3 was significantly decreased compared with that of the CLP group (*P* < 0.05). In addition, MaR1 treatment inhibited STAT3 phosphorylation in septic mice, which is consistent with changes in IL-6 levels after MaR1 treatment.

**Figure 6:**
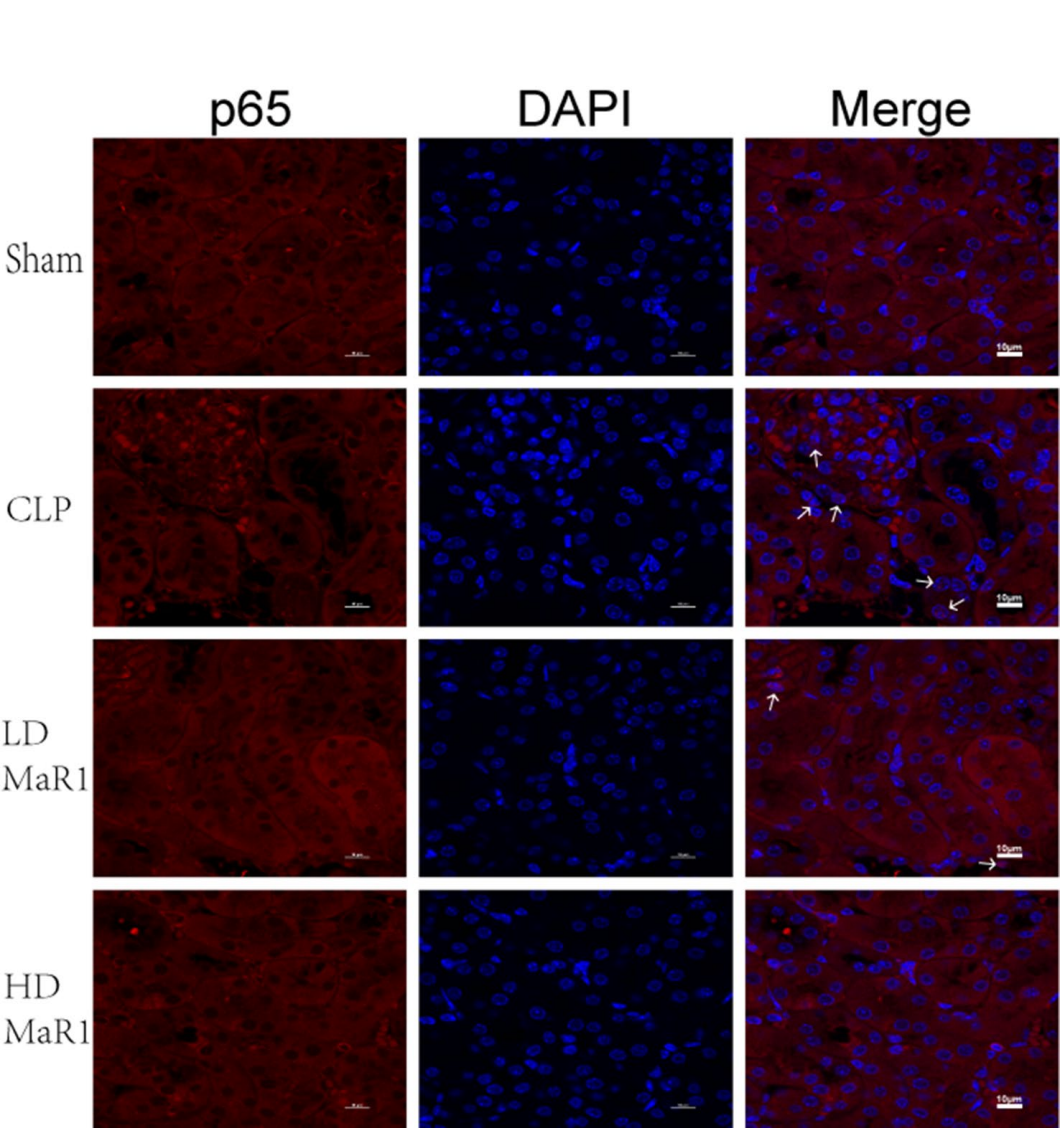
MaR1 inhibits p65 nuclear translocation in the kidney of sepsis mice. Representative immunofluorescence staining of kidney tissue at 24 h after CLP in each group of mice, and white arrow indicates p65 into the nucleus, magnification 1,000×. MaR1, Maresin 1; CLP, cecal ligation and puncture.

**Figure 7:**
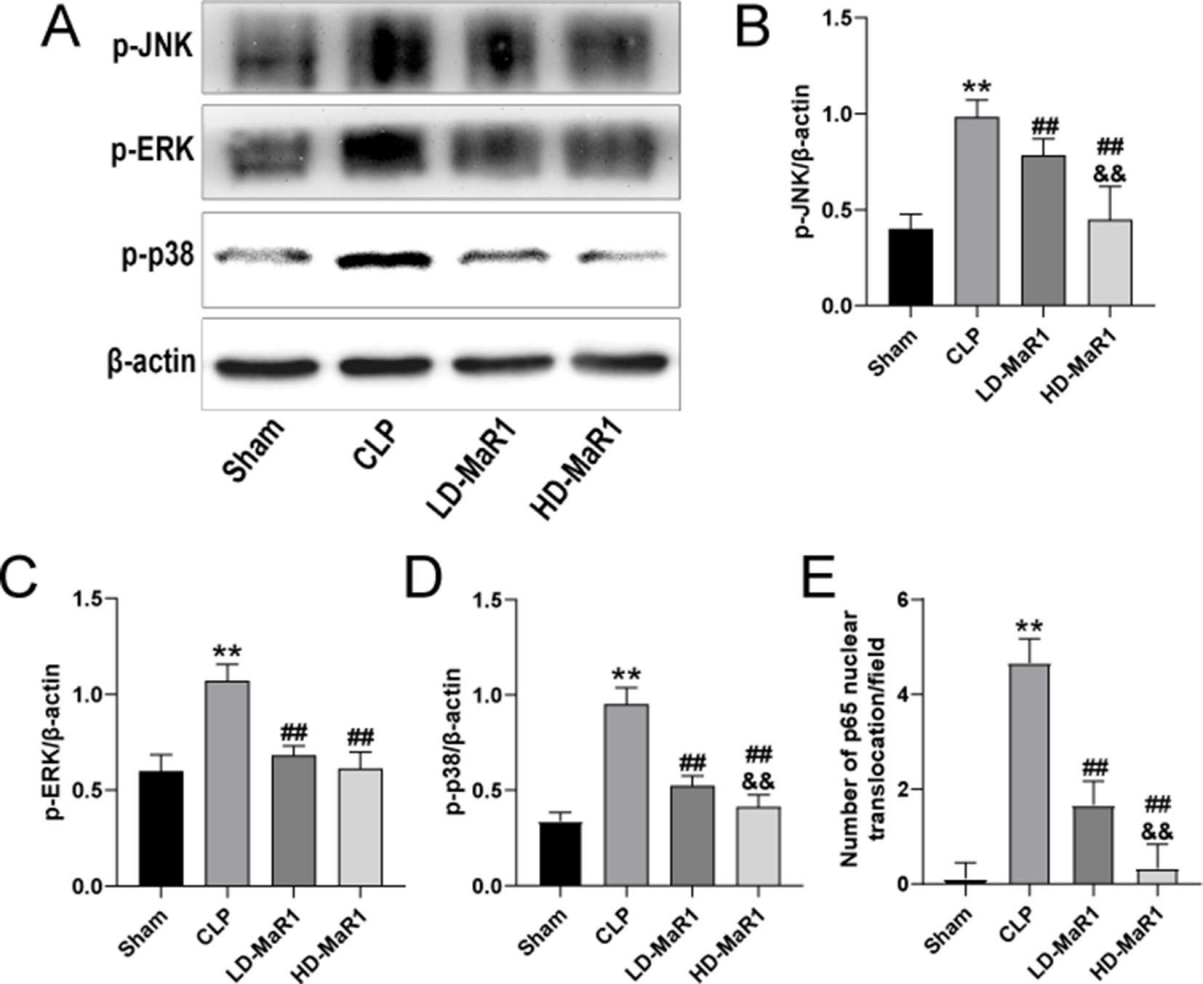
MaR1 inhibits MAPK pathway activation and p65 nuclear translocation in the kidney of sepsis mice. The expressions of p-JNK, p-ERK, and p-p38 in renal tissues of mice in each group were analyzed by Western blot **(A)**. The p-JNK **(B)**, p-ERK **(C)**, and p-p38 **(D)** protein levels normalized by β-actin. **(E)** Nuclear translocation of p65 count/field. Data are shown as mean ± SD, *n* = 9. **P 0.01 vs. sham group; ^##^ *P* 0.01 vs. CLP group; ^&&^ *P* 0.01 vs. LD-MaR1 group. MaR1, Maresin 1; MAPK, mitogen-activated protein kinase; CLP, cecal ligation and puncture; LD-MaR1, MaR1 low-dose group.

In addition, we performed immunofluorescence staining on kidney tissue samples to detect the nuclear translocation of p65. After 24 h of CLP, p65 into the nucleus significantly increased compared with the sham group (*P* < 0.01), while MaR1 treatment showed dose-dependent inhibition of p65 nuclear transfer (**Figures 6** and **7E**).

### MaR1 Inhibits MAPK Signaling Pathway in CLP-Induced AKI

The mitogen-activated protein kinase (MAPK) chain is one of the important pathways in eukaryotic signaling networks and plays an important role in cell growth and differentiation, and stress responses such as inflammation and apoptosis. In our study, the JNK, ERK, and p38 signaling pathways were analyzed in order to explore the protective mechanism of MaR1 on SA-AKI. As shown in **Figures 7A–D**, the phosphorylation of JNK, ERK, and p38 in the renal tissues of CLP group was significantly increased compared with the sham group (*P* < 0.01). However, MaR1 significantly inhibited phosphorylation of JNK, ERK, and p38 in renal tissue of mice after CLP (*P* < 0.01).

## Discussion

Inflammation is an important defense mechanism against the infection and injury of the body, but excessive uncontrolled inflammatory reaction can cause non-specific killing of self-organized cells (Chiurchiù et al., 2018). To limit the excessive development of inflammation and promote the timely regression of inflammation, the body will internally produce a series of anti-inflammatory and pro-resolving lipid mediators, which are also known as the “brake signal” of inflammation. MaR1 is one of the newly discovered anti-inflammatory and pro-resolving mediators, which can inhibit neutrophil infiltration and adhesion, enhance macrophage phagocytosis in apoptotic neutrophils and necrotic cells, down-regulate the production of inflammatory mediators, inhibit the activation of NF-κB, increase the synthesis of regulatory T cells, and improve the level of intracellular cyclic adenosine monophosphate to exhibit a powerful anti-inflammatory and pro-resolving effects in multiple animal models (Serhan et al., 2009; Serhan, 2014;Yang et al., 2019).

The kidney is one of the organs most frequently involved in sepsis, and AKI is an independent risk factor for the increased risk of death in patients with sepsis (Lopes et al., 2009). Therefore, strengthening the prevention and treatment of AKI in patients with sepsis is of great significance to improve the prognosis of patients. Our study used CLP model of mice to explore the protective effect of MaR1 on SA-AKI and its related mechanisms and to provide a basis for the treatment of the SA-AKI in clinical practice. Our experimental results show that the kidney tissue damage of the CLP group was obvious, and the levels of Cr and BUN in the serum were elevated, indicating that the model of SA-AKI was successfully established. The deterioration of renal function in the CLP model was improved after MaR1 treatment, and the pathological damage of renal tissue was significantly reduced, indicating that MaR1 has a significant protective effect on SA-AKI. In addition, MaR1 reduced systemic inflammation and mortality in sepsis mice.

Many experimental evidences show that an excessive neutrophil recruitment into the kidney during sepsis plays an important role in the deterioration of renal function (Castoldi et al., 2012; Herter et al., 2014), while inhibition of neutrophil migration can attenuate sepsis-induced AKI (Li et al., 2017). In classical immunology, neutrophils are one of the most important components of innate immunity. These cells migrate to the infected site under the action of chemokines to exert antibacterial effects, which is essential for the control of local infections and the prevention of systemic spread of infection (Ermert et al., 2009; Kolaczkowska and Kubes, 2013). However, in severe sepsis, the ability of neutrophils to shift to the site of infection is inhibited, and the deterioration of infection cannot be effectively and timely controlled. Instead, more neutrophils are concentrated in vital organs such as the lungs, kidneys, and heart, which not only caused the obstruction of capillary vessels and affected the blood supply of tissues but also released various enzymes and inflammatory mediators to cause the damage of related tissues and cells and eventually developed into multiple organ failure (Sônego et al., 2016). Therefore, to some extent, inhibiting neutrophil infiltration to important organs is of great significance in reducing organ damage in severe sepsis. Previously, it has been demonstrated in the mice model of LPS-induced acute lung injury that MaR1 alleviates LPS-induced lung injury, which is associated with inhibition of neutrophil infiltration (Gong et al., 2015). Our immunohistochemical results showed that MaR1 significantly reduced the number of ly-6G-positive cells in renal tissue in a dose-dependent manner, and the detection of MPO activity in renal tissue homogenate further demonstrated the decreasing trend of neutrophils infiltration after treatment with MaR1; interestingly, this change was consistent with the trend of renal damage reduction following MaR1 administration. Consistent with our findings, a similar study has also shown that aspirin-triggered resolvin D1 (AT-RvD1) attenuates endotoxin-induced acute kidney damage by inhibiting renal neutrophil infiltration (Chen et al., 2014). Of course, the mechanism behind MaR1 inhibition of neutrophil infiltration may be multifaceted, involving a reduction in chemokines/cytokines, changes in the expression of cell adhesion molecules, and/or a direct effect on neutrophils (Marcon et al., 2013).

Although the pathogenesis of SA-AKI may be multifaceted, the inflammatory cascade is one of the important factors that promote the development of SA-AKI (Chen et al., 2014; Qiu et al., 2018; Tang et al., 2018; Poston and Koyner, 2019). In this study, we further explored the protective effect of MaR1 on SA-AK by inhibiting inflammatory signaling pathways. NF-κB is present in almost all cells, and the family is composed of five structurally related members (p50, p52, p65, RelB, and c-Rel) (Sun et al., 2013). It can regulate the expression of various cytokine genes by multi-directional transcription and plays a key role in the inflammatory response and immunity. At rest, NF-κB binds to the inhibitor IκB to form an inactive trimer existing in the cytoplasm, with no function of regulating gene transcription. When the cells are stimulated, they induce the activation of NF-κB kinase, which degrades IκB and phosphorylates the p65 subunit and transfers it into the nucleus. It participates in the regulation of the expression of a series of inflammatory cytokines (e.g., TNF-α, IL-1β, IL-6, and IL-8) by binding to specific DNA fragments (Mattioli et al., 2004; Liu et al., 2017). It is worth noting that TNF-α is a downstream molecule of the NF-κB pathway, but it can also activate the NF-κB pathway to promote the expression of other inflammatory cytokines and thereby expand the inflammatory cascade (Sanchez-Niño et al., 2010). Previous studies have described the role of MaR1 in inhibiting NF-κB activation in different animal models (Marcon et al., 2013; Qiu et al., 2019; Yang et al., 2019). In the study, we examined the expression of p-p65, a key active factor downstream of the NF-κB pathway, in the kidney tissue by Western blotting; meanwhile, the nuclear translocation of p65 in renal tissue cells was detected by immunofluorescence assay. The results are consistent with other studies, confirming that MaR1 treatment effectively inhibited the phosphorylation of p65 and decreased the nuclear translocation of p65. It is noteworthy that the inflammatory cytokines in renal tissue homogenate have been altered with the inhibition of NF-κB after MaR1 administration. The ELISA results showed that MaR1 treatment significantly reduced the levels of pro-inflammatory cytokines (TNF-α, IL-1β, and IL-6) in sepsis mice and increased the production of the anti-inflammatory cytokine IL-10, suggesting that the protective effect of MaR1 on SA-AKI may, at least in part, be related to the inhibition of NF-κB pathway.

The STAT proteins are widely expressed in different types of cells and tissues and are involved in the regulation of various physiological functions such as cell growth, differentiation, and apoptosis and are closely related to inflammation, tumors and immune response (Leeman et al., 2006). IL-6 is an important player in the acute phase of inflammation and is closely related to the mortality of sepsis (Kamimura et al., 2003; Wang et al., 2009). The STAT3, a major downstream signaling target for intracellular signaling of IL-6, plays a key role in IL-6-mediated inflammation and immunity (Fang et al., 2013). Our results indicate that MaR1 can exert renal protection in SA-AK by reducing IL-6 expression and phosphorylation of STAT3. Interestingly, the AT-RvD1, the lipid mediator also derived from DHA, in inhibiting LPS-induced STAT3 phosphorylation and blocking IL-6-mediated signaling pathways of renal tissues to attenuate LPS-induced renal damage has long been reported (Chen et al., 2014). The MAPK, including the JNK, ERK, and p38, is an important transmitter of signal transduction from the cell surface to the interior of the nucleus. It can be activated by a variety of stimulating factors or signaling molecules outside the cell, and then signal transduction through a three-level kinase cascade to amplify the external stimulus signal, and transduction into the cytoplasm or nucleus, driving downstream cytokines involved in a variety of physiological and pathological processes, such as cell proliferation and differentiation, inflammation, apoptosis, and other stress responses, plays an important role in the regulation of various cellular activities (Johnson and Lapadat, 2002; Koul et al., 2013). Previous studies have shown that the MAPK pathway plays an important role in regulating renal inflammation and cell death (Basu and Krishnamurthy, 2010). Our results indicate that MaR1 can significantly inhibit CLP-induced JNK, ERK, and p38 phosphorylation in the kidney, suggesting that the MAPK pathway may be involved in the protective effect of MaR1 on SA-AKI.

It is well known that macrophages are an important component of the innate immune system and play an important role in the development and regression of inflammation. In terms of function, macrophages can be classified into the classical M1 type, the non-classical M2 type, and the intermediate type in the local microenvironment, in which M1 macrophages exhibit obvious pro-inflammatory actions, while M2 macrophages are more prone to pro-resolving effects (Gordon and Taylor, 2005; Martinez et al., 2009). Marcon et al. (2013) pointed out that the pro-resolving effects of MaR1 on experimental colitis may be related to MaR1 promoting macrophage transition from M1 to M2 type. However, whether the protective effect of MaR1 on SA-AKI involves the intervention of MaR1 on different phenotypes of macrophages will be the direction of our future research. Qiu et al. (2019) found that MaR1 can alleviate renal ischemia/reperfusion injury in mice by exerting anti-oxidation effect, but whether the protective effects of MaR1 on SA-AKI is related to anti-oxidation is still unclear. In addition, the binding receptors in which MaR1 plays a role in SA-AKI remain unknown. Therefore, in order to more fully clarify the specific protection mechanism of MaR1 for SA-AK and answer the mode-of-action of MaR1, more research is needed in the future.

## Conclusion

Our results confirmed that MaR1 can reduce mortality and effectively reduce acute kidney damage of sepsis mice, and the mechanism may be through decreased neutrophil infiltration and inhibition NF-κB/STAT3/MAPK activation and thereby reducing pro-inflammatory cytokines and increasing anti-inflammatory cytokine levels. The current conclusion supports MaR1 as a potential new treatment for SA-AK, and of course a large number of clinical studies are needed in the future to further confirm its effectiveness.

## Data Availability Statement

The datasets analyzed in this manuscript are not publicly available. Requests to access the datasets should be directed to the corresponding author HX.

## Ethics Statement

The animal study was reviewed and approved by The Animal Committee of Tongji Medical College, Huazhong University of Science and Technology.

## Author Contributions

SS, JiaW, JinW, and FW performed experimental operations and contributed to data collection. SS and HX contributed to data analysis. SS contributed to manuscript writing. HX and SY contributed to the design of the experiments and revised the article. All authors read and approved the final manuscript.

## Funding

This study was supported by grants from the National Natural Science Foundation of China (no. 81701887).

## Conflict of Interest

The authors declare that the research was conducted in the absence of any commercial or financial relationships that could be construed as a potential conflict of interest.
